# The oncologists' and radiologists' PET

**DOI:** 10.1038/sj.bjc.6601190

**Published:** 2003-07-15

**Authors:** Robin A Weiss

In this issue, Professor Karen Facey and colleagues publish an important Commentary (p 224) on the use of positron emission tomography (PET) imaging in cancer management. As they point out, any novel health technology tends to generate calls for immediate implementation by clinical enthusiasts, but the case for investment should be based on a clear evidence of benefit to patients (rather than to doctors) as well as cost benefit. PET using fluorine-18 deoxyglucose has given oncologists insight into tumour metabolism in addition to imaging, and it is a superb clinical research tool. But should PET imaging become routine?

The Commentary, based on a recent Health Technology Assessment report, is timely for the UK because the Royal Colleges Intercollegiate Standing Committee on Nuclear Medicine earlier this year recommended the development and expansion of PET imaging. This Committee's conclusions are almost entirely devoted to the logistics of implementation, essentially following American assumptions, without critical appraisal of their utility. That is why the Health Technology Assessment on PET is so important.

The authors make two salient points. First, they show that evidence of accuracy is not directly translatable into cost benefit, and therefore patient benefit should be recognised as a more important end point than potential cost saving. Indeed, it is surprising that although PET imaging was introduced some 20 years ago and has accrued more than 1000 research publications, patient outcome has not been adequately addressed. Second, the novel decision modelling scheme developed by these investigators, which includes the handling of uncertainty, may in this type of situation provide more useful evidence for policy making than a randomised controlled clinical trial. No doubt, oncologists will initially be reluctant to acknowledge this paradigm shift.

